# Supplementary material for: A Multi-Factorial Observational Study on Sequential Fecal Microbiota Transplant in Patients with Medically Refractory Clostridioides difficile Infection
Source: Cells. 2021 Nov 19;10(11):3234. doi: 10.3390/cells10113234 (PMC8624539; doi:10.3390/cells10113234)
Supplement: Supplementary file 1 [file cells-10-03234-s001.zip › Supplementary materials.pdf]

Supplementary Materials

Supplementary Methods

DNA extraction for 16S rRNA gene sequencing:
DNA was extracted from faecal samples (~250 mg of stool) via the DNeasy PowerSoil Kit (Qiagen, UK) using manufacturer's instructions, but with the addition of a bead-beating step at speed 8 for 3 min via use of a Bullet Blender Storm (Chemobio Ltd, UK)(Mullish et al., 2018). Extracted DNA was quantified using a Qubit 2.0 Fluorometer (ThermoFischer Scientific, UK), and stored at -80°C pending downstream assays.

Metabolomic analysis:

Ultra-performance liquid chromatography-mass spectrometry (UPLC-MS) profiling and analysis of fecal bile acids
The protocols used for faecal extract preparation and data accumulation were as previously described (15, 82). In addition, mass spectrometry data was analyzed using peakPantheR, an automated pipeline for the detection, integration and reporting of predefined features across a large number of mass spectrometry data files (https://github.com/phenomecentre/peakPantheR) (Wolfer et al., 2020).

Gas chromatography-mass spectrometry (GC-MS) for the detection, identification and quantification of short chain fatty acids in feces and serum
Targeted GC-MS was performed using adaptation of previously described protocols for the analysis of samples of stool (18, 84) and serum (Moreau et al., 2003). Samples analysis was performed on an Agilent 7890B GC system coupled to an Agilent 5977A mass selective detector (Agilent, USA). Analysis of data was performed using MassHunter software (Agilent), with SCFA concentrations also being integrated from a freshly prepared calibration curve for each standard to enable quantification.
NB: References 82-85 only appear in Supplementary Materials

Stimulation of PBMCs to induce cytokine production by CD4 T cells
PBMCs in RPMI medium containing FCS (10%) and supplemented with glutamine-streptomycin-penicillin (1 x 10^{6} /ml) were stimulated with PMA (50ng/mL, Sigma Aldrich) Ionomycin (100ng/mL; Sigma Aldrich) and Brefeldin A (10µg/ml; Sigma Aldrich) for 4 hr at 37°C. Post stimulation, the cells were surface-stained (anti-human CD3, anti-human CD4), fixed with Reagent A (Thermo Fischer Scientific) for 30 min, followed by a wash and permeabilization with permeabilization reagent (Thermo Fischer Scientific), and stained with a combination of antibodies including anti-human IL17 APC, anti-human IFNγ PE, anti-human IL4 APC, anti-human IL10 PE, anti-human IL6 PE and anti-human TNFα FITC for 30 min at 4°C. This

step was followed by two washes with PBS. Samples were acquired using a Cyan ™ ADP flow cytometer (Dako).

Staining for toxin expressing immune cells

PBMCs in PBS (1 x 10^6 cells/mL) were incubated with anti-*C.difficile* Toxin A therapeutic antibody – PE (100µg, Creative Biolabs) or anti-*C.difficile* Toxin B therapeutic antibody – PE (100µg , Creative Biolabs) for 1 hr on a rocker at 4°C. Post washing with PBS, cells were stained with combinations of antibodies for 30 min at 4°C, followed by a wash and 15 min with 1 % formaldehyde solution (Thermo Fischer Scientific). Samples were acquired using a Cyan ™ ADP flow cytometer (Dako).

Immunostaining via flow cytometry

T cell subset distribution: CD3, CD4, PTK7, CCR7, CD45RA (APC)

T cell gut homing potential: CD3, CD4, CD45RA (PEcy5.5), CCR9, Integrin

T cell senescence markers: CD3, CD4, CD8, NKG2D, CD28, CD57

T cell activation: CD3, CD4, CD8, CD69

Regulatory T cells: CD3, CD4, CD25, Foxp3

Follicular helper T cells: CD3, CD4, CXCR5, bcl6

Dendritic cell subset distribution: lineage cocktail, HLADR, CD11c, CD80, CD86

B cell subset distribution: CD19, IgD, CD27, CD138

B cell gut homing potential: CD19, CD27, CCR9, Integrin

Regulatory B cells: CD19 (FITC), CD24, CD38, CCR9

Natural Killer cell subsets: CD3, CD56, CD69, NKG2D

List of antibodies used in flow cytometry

Antibody	Supplier	Clone
**Surface markers**		
anti-human CD3-PEcy7	Thermo Fischer	UCHT1
anti-human CD4 Violet	Thermo Fischer	RPA-T4
anti-human CD8 PE	Immunotools	UCHT4
anti-human CCR7 FITC	R and D systems	150503
anti-human CD45RA APC	Biolegend	HI-100
Anti-human CD45RA Pecy5.5	Biolegend	H1101
anti-human CCR9 PE	R and D systems	MAB179
anti-human Integrin APC	Biolegend	F1B504
anti-human CD28 APC	B D Biosciences	CD28.2
anti-human CD57 FITC	Thermo Fischer	HCD57
Anti-human CD69 APC	Thermo Fischer	H1.2F3
Anti-human CXCR5 APCcy7	Biolegend	J25204
anti-human PTK7 PE	Miltenyi Biotech	188B
anti-human CD19 PE	Thermo Fischer	HIB19
anti-human CD27 Violet	Thermo Fischer	O323
Anti-human CD138 APC	Thermo Fischer	DL101
anti-human CD56 PE	Miltenyi Biotech	AF12 7H3
anti-human IgD-FITC	Thermo Fischer	1A6-2
anti-human CD24-FITC	Thermo Fischer	eBioSN3
anti-human CD38-PEcy7	Thermo Fischer	HIT2
anti-human lineage cocktail pacific blue	Biolegend	
anti-human HLADR PEcy7	Biolegend	L243
Anti-human CD11c APC	Biolegend	3.9
Anti-human CD80 FITC	BD Biosciences	L307.4
Anti-human CD86 PE	BD Biosciences	2331
**Transcription factors**		
Anti-human Foxp3 PE	Thermo Fischer	PCH101
Anti-human bcl6 APC	Thermo Fischer	BCL-UP
**Intracellular cytokine antibodies**		
Anti-human IL17 APC	Thermo Fischer	eBio640EC17
Anti-human IFN γ PE	Biolegend	B27
Anti-human IL10 PE	Thermo Fischer	JE53-9D7
Anti-human IL4 APC	Biolegend	8D48
Anti-human TNF FITC	BD Biosciences	Mab 11
Anti-human IL6 PE	Biolegend	MQ2-13A5
**Isotype controls**		
Mouse IgG1 PE	Biolegend	MPC11
Mouse IgG1 FITC	Thermo Fischer	eBM2a
Mouse IgG2b Violet	Biolegend	MOPC21
Mouse IgG1 APC	Biolegend	MOPC21
Mouse IgG1PEcy7	Thermo Fischer	P3.6.281
Mouse IgG2b PEcy5.5	Biolegend	MPC11

Microarray methods

Antigens and bacterial lysates

For the control antigens, *candida albicans* cell wall and cytoplasmic antigen preparations were obtained from Jena Bioscience GmbH, Germany. CMV purified antigen (strain AD-169) was obtained from The Native Antigen Company (2BScientific, Oxford, UK) (CMP-HP-50UG). EBV viral capsid antigen GP125 (this antigen was used at 30µg/mL) was obtained from Meridian Life Sciences (Tebu-Bio, Cambs.UK). Tetanus toxoid was obtained from the National Institute for Biological Standards and Controls, UK.

For the test antigens, we used highly purified *C. difficile* antigens including *C. difficile* whole toxins TcdA (toxin A) and TcdB (toxin B); toxinotype 0, strain VPI 10463, ribotype 087, kindly gifted by Dr April Roberts, Public Health England), available surface layer protein extracts (SLP) previously purified from three *C. difficile* strains (ribotypes 001, 002, 027, gifted from Professor Christine Loscher, Dublin City University). Lysates of these ribotypes were prepared from freshly grown overnight broth cultures (BHIS). Briefly, 1.5 mL culture was centrifuged at 14,000 g for 10 min and the supernatant discarded. The pellet was resuspended in 300 µL of BugBuster® HT Protein Extraction Reagent (Novagen) and incubated for 20 min at room temperature. Lysates were frozen until required. On each array, a series of 10-point dilutions of purified human immunoglobulin matching the tested isotype were printed as a calibrator to generate a standard curve.

Microarray procedure

Ten microliters of each diluted target antigen, control antigen and landing light (corner marker) were added into wells of a 384 well, full skirted PCR plate (Eppendorf twin.tec) and microarrays were printed in a 8x2 arrangement onto aldehyde-activated 75x25mm glass microscope slide surface, using an Apogent Microgrid 610 arraying robot (Apogent, USA) with solid arraying pins at a humidity of 70%, with double spotting of each feature. Arrays were rested overnight at room temperature and processed by adding a 16 well Proplate seal and clamps (Grace Biolabs, Oregon, USA). Arrays were blocked by the addition of 100µL Intercept PBS blocking buffer (Li-Cor Biosciences UK Ltd, Cambridge, UK) and incubation for 1 h at room temperature with shaking. Each well was washed 3 times with 100uL of PBS, 0.05% Tween-20 (PBST), inverting and tapping out all liquid after each wash. One hundred microliters of 1:50 diluted serum in Intercept buffer was added to each well and incubated for 1 h at room temperature. Three wells were used for each serum sample to allow measurement of IgG, IgA and IgM responses. Each well was washed 3 times with 100µL of PBS, 0.05% Tween-20 (PBST), inverting and tapping out all liquid after each wash. One hundred microliters of appropriate biotinylated secondary antibody solutions were diluted in Intercept blocking buffer as stated (anti-human IgG (1:20,000), anti-Human IgA (1:10,000) and anti-human IgM (1:10,000), all from Thermofisher Scientific, UK. The diluted biotinylated antibodies were applied to separate wells for each antibody

class examined and incubated for 1 h. After 3 more washes in PBST as before, each well was amplified by incubation with 100µL of a streptavidin-IR800 dye conjugate, diluted 1:20,000 in PBST+0.01% SDS(Li-Cor BioSciences UK) for 30 min. The wells were washed 3 times as before with PBST. Each well was incubated with 100mL of biotinylated-anti-streptavidin (Vector Laboratories Inc.) diluted 1:2000 in PBST. After a further three washes in PBST, each well was incubated with 100µL of a streptavidin-IR800 dye conjugate, diluted 1:20,000 in PBST+0.01% SDS (Li-Cor BioSciences UK) for 30 minutes, washed 3 times in PBST. The incubation cassette was dismantled and slides rinsed briefly in distilled water and dried by centrifugation at 600g for 10 seconds using a Labnet Slide Spinner (Sigma-Aldrich, UK). Fluorescent data from each microarray was captured using a Li-Cor Odyssey SA near-infra-red scanner, set to 20µm resolution and a sensitivity of 10.5 for the 800nm channel, 7 for the 680 channel. Microarray Images were analysed used Axon Genepix Pro software, version 6.8 to determine feature (spot) intensity and morphology and local background for each antigen feature. Further analysis was performed using Microsoft Excel and Graphpad Prism v8.

RNA isolation, TCR library preparation and sequencing

Briefly, cDNA synthesis was performed using SMARTScribe reverse transcriptase (Clontech, Takara) using primers for the TCRα and TCRβ constant region. A unique molecular identifier (UMI), and a sample barcode of 6 nucleotides, were introduced via template-switching. cDNA synthesis was carried out for 60 minutes at 42°C. cDNA was treated with Uracil DNA-Glycosylase (UDG, from New England Biolabs) and incubated for 30 minutes at 37°C. Samples were subsequently purified with the QIAquick PCR purification kit (Qiagen) and eluted in 50 µl deionized water. Purified cDNA was amplified with 2 consecutive PCRs, respectively 18 and 12 cycles, with purification after each PCR using MagSi-NGSprep Plus (MagnaMedics). Illumina compatible adapters and sample-specific barcodes were added during the second PCR. Quality and concentration of the libraries were measured with TapeStation D1000 (Agilent) and Qubit (Thermofisher).

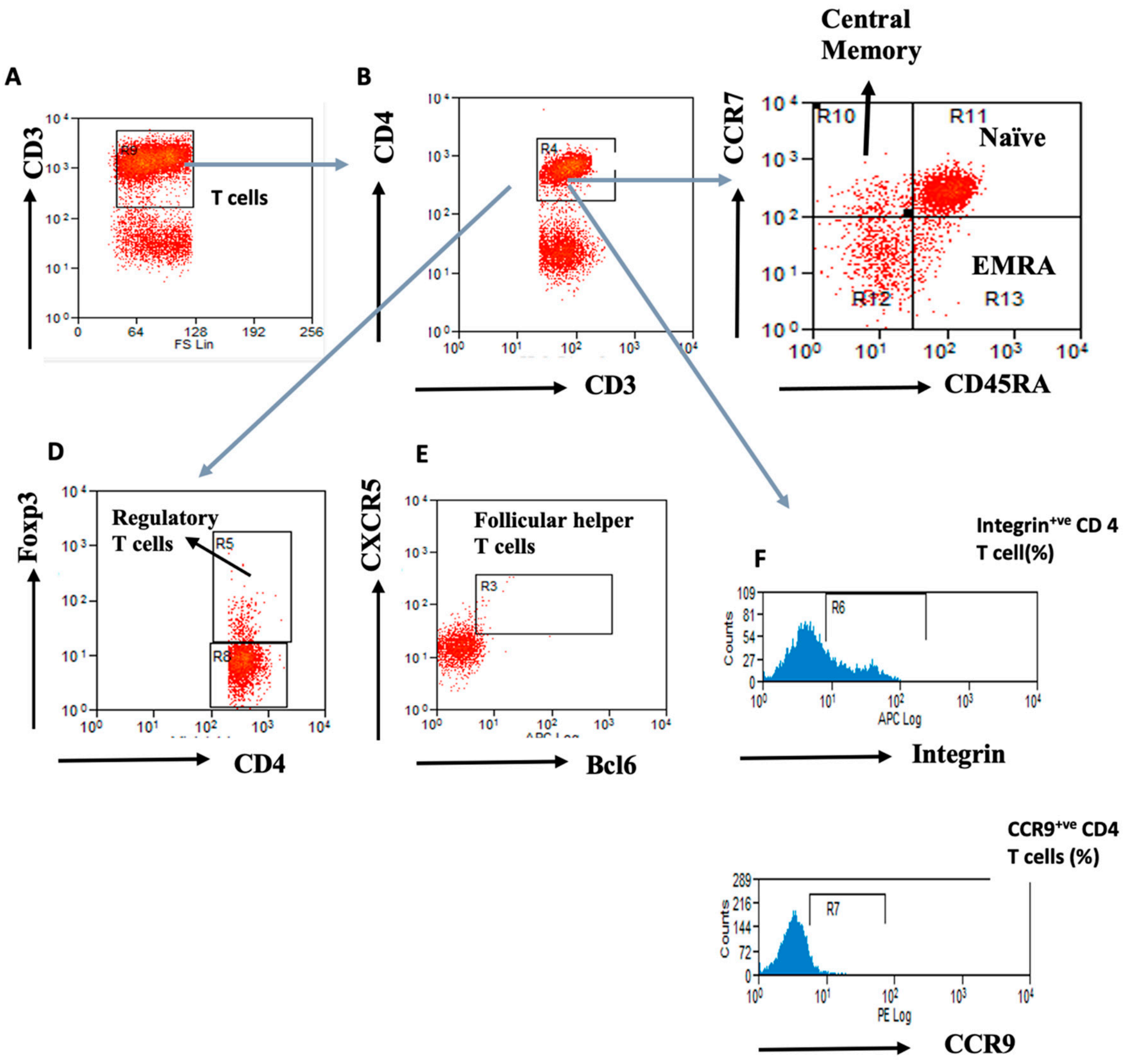

Supplementary Figure S1. Gating strategy for CD4 T cell subsets. Gating strategy for CD4 T cell subsets. (A) CD3^{+ve} T cells (B) CD4^{+ve} CD3^{+ve} T cells (C) Naïve, central memory, effector memory, effector memory CD4 T cells have been identified on the basis of CCR7 and CD45RA expression (D) CD4^{+ve} CD3^{+ve} T cells Foxp3^{+ve} regulatory T cells (E) CXCR5^{+ve} bcl6^{+ve} CD3^{+ve} CD4^{+ve} follicular helper T cells. (F) expression of CCR9 and Integrin on CD4 T cells.

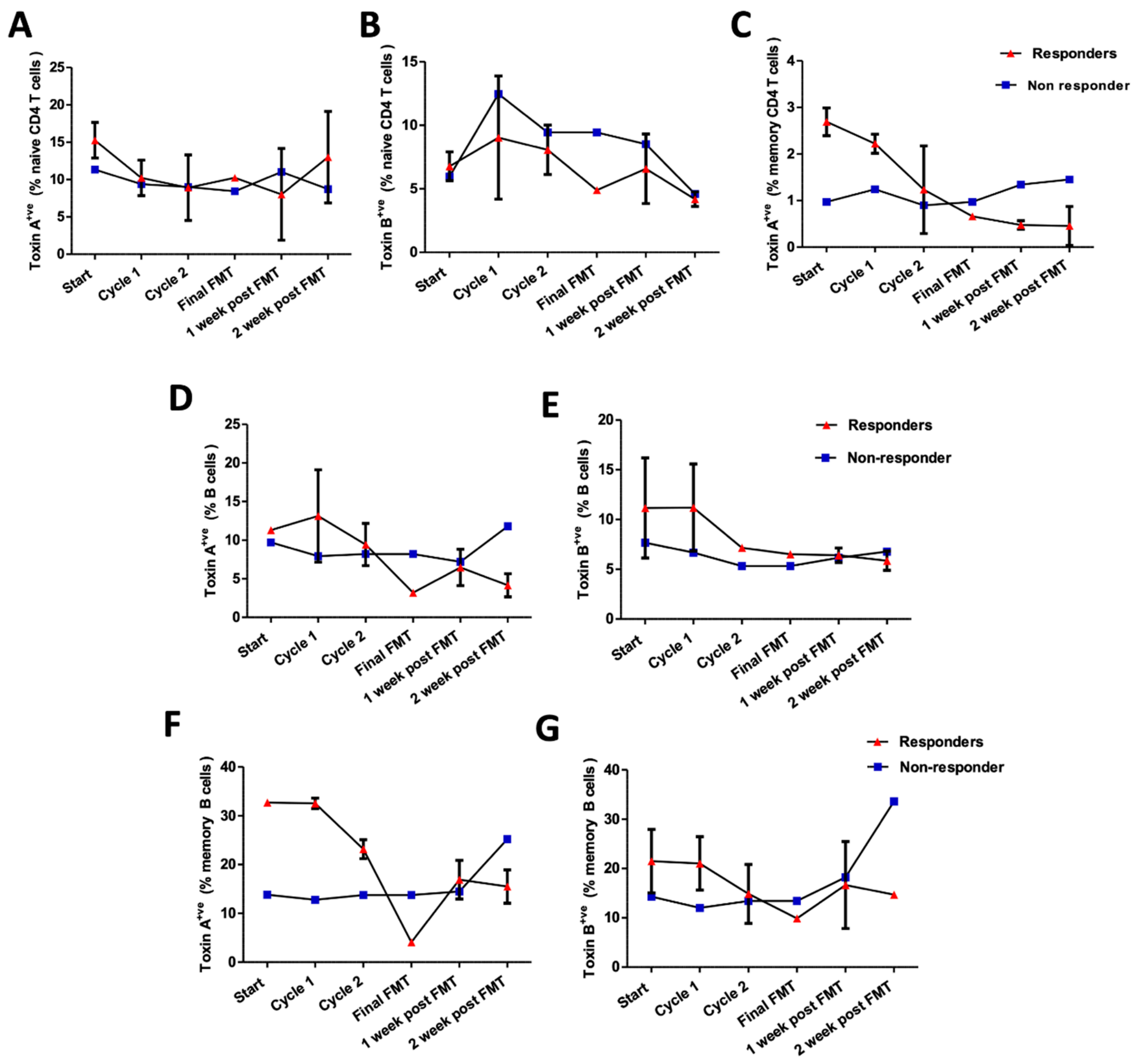

Supplementary Figure S2. Toxin expressing T and B cells in patients with severe or fulminant *Clostridioides difficile* infection in relation to sequential FMT. Percentage of peripheral (A) Toxin A^{+ve} naïve CD4 T cells (B) Toxin B^{+ve} naïve CD4 T cells (C) Toxin A^{+ve} memory CD4 T cells (D) Toxin A^{+ve} B cells (E) Toxin B^{+ve} B cells (F) Toxin A^{+ve} memory B cells (G) Toxin B^{+ve} memory B cells in responders and one non-responder patient at the screening, post FMT cycle 1, post FMT Cycle 2, post final FMT cycle, 1 week and 2 weeks after FMT. The data are mean ± S.D for the 2 responders.

A

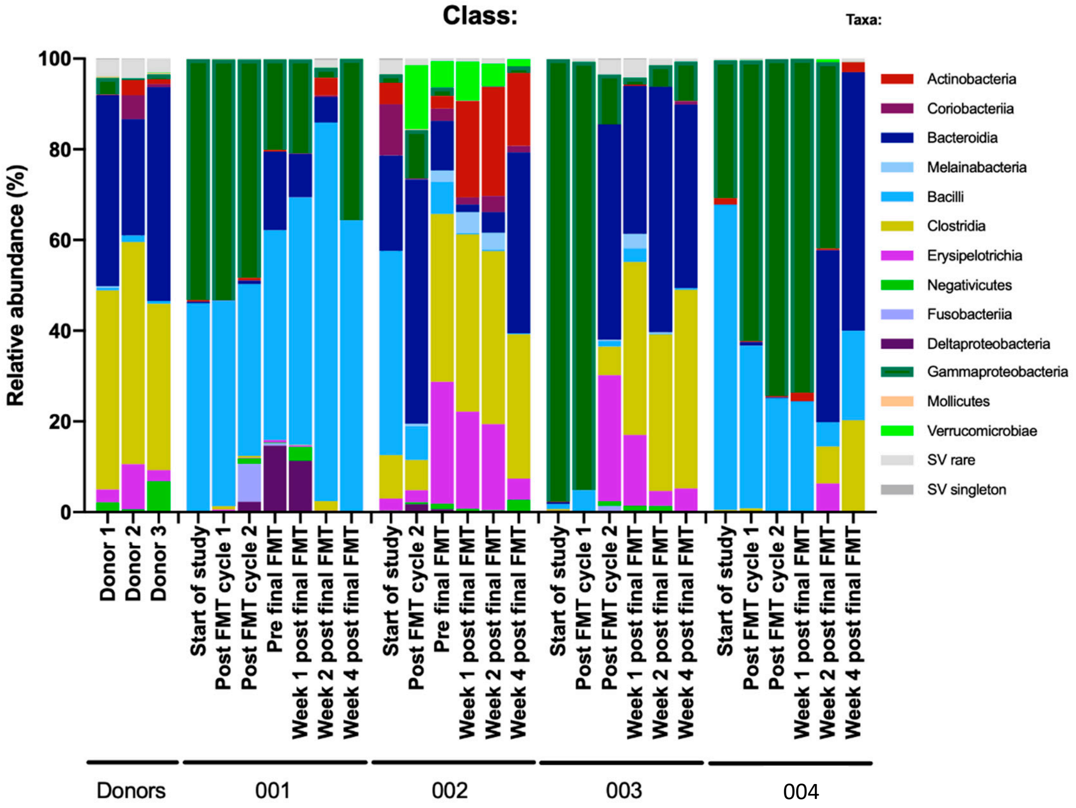
Class:
Taxa:
Relative abundance (%)
100
80
60
40
20
0
Actinobacteria
Coriobacteriia
Bacteroidia
Melainabacteria
Bacilli
Clostridia
Erysipelotrichia
Negativicutes
Fusobacteriia
Deltaproteobacteria
Gammaproteobacteria
Mollicutes
Verrucomicrobiae
SV rare
SV singleton
Donor 1
Donor 2
Donor 3
Start of study
Post FMT cycle 1
Post FMT cycle 2
Pre final FMT
Week 1 post final FMT
Week 2 post final FMT
Week 4 post final FMT
Donors
001
002
003
004

B

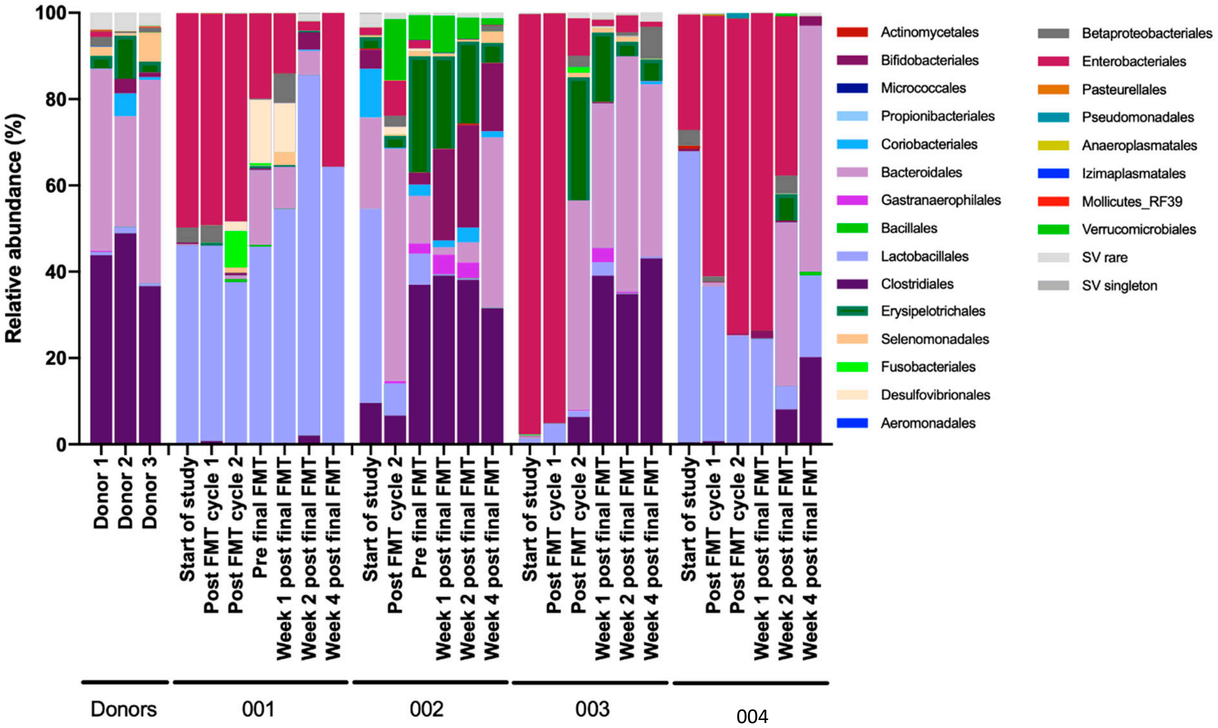
Relative abundance (%)
100
80
60
40
20
0
Actinomycetales
Bifidobacteriales
Micrococcales
Propionibacteriales
Coriobacteriales
Bacteroidales
Gastranaerophilales
Bacillales
Lactobacillales
Clostridiales
Erysipelotrichales
Selenomonadales
Fusobacteriales
Desulfovibrionales
Aeromonadales
Betaproteobacteriales
Enterobacteriales
Pasteurellales
Pseudomonadales
Anaeroplasmatales
Izimaplasmatales
Mollicutes_RF39
Verrucomicrobiales
SV rare
SV singleton
Donor 1
Donor 2
Donor 3
Start of study
Post FMT cycle 1
Post FMT cycle 2
Pre final FMT
Week 1 post final FMT
Week 2 post final FMT
Week 4 post final FMT
Donors
001
002
003
004

C

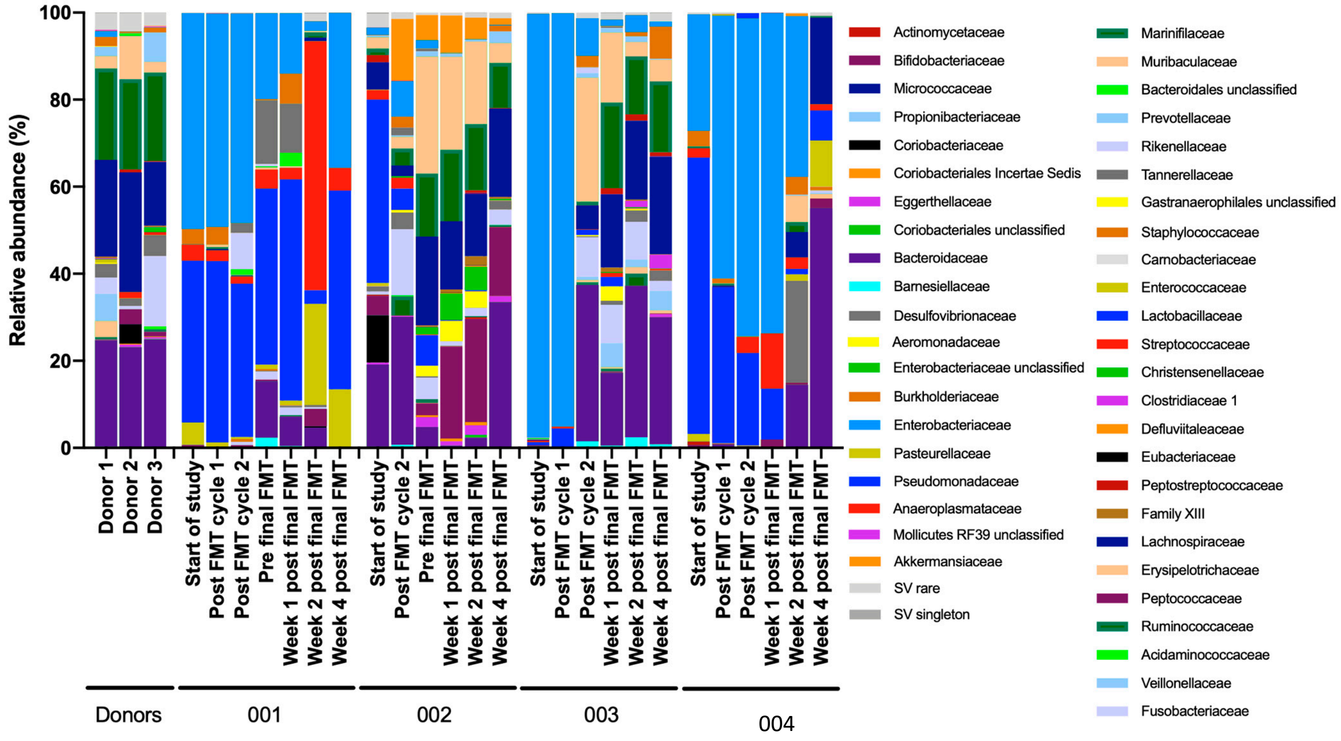
Relative abundance (%)
100
80
60
40
20
0
Donor 1
Donor 2
Donor 3
Start of study
Post FMT cycle 1
Post FMT cycle 2
Pre final FMT
Week 1 post final FMT
Week 2 post final FMT
Week 4 post final FMT
Donors
001
002
003
004
Actinomycetaceae
Bifidobacteriaceae
Micrococcaceae
Propionibacteriaceae
Coriobacteriaceae
Coriobacteriales Incertae Sedis
Eggerthellaceae
Coriobacteriales unclassified
Bacteroidaceae
Barnesiellaceae
Desulfovibrionaceae
Aeromonadaceae
Enterobacteriaceae unclassified
Burkholderiaceae
Enterobacteriaceae
Pasteurellaceae
Pseudomonadaceae
Anaeroplasmataceae
Mollicutes RF39 unclassified
Akkermansiaceae
SV rare
SV singleton
Marinifilaceae
Muribaculaceae
Bacteroidales unclassified
Prevotellaceae
Rikenellaceae
Tannerellaceae
Gastranaerophilales unclassified
Staphylococcaceae
Carnobacteriaceae
Enterococcaceae
Lactobacillaceae
Streptococcaceae
Christensenellaceae
Clostridiaceae 1
Defluviitaleaceae
Eubacteriaceae
Peptostreptococcaceae
Family XIII
Lachnospiraceae
Erysipelotrichaceae
Peptococcaceae
Ruminococcaceae
Acidaminococcaceae
Veillonellaceae
Fusobacteriaceae

D

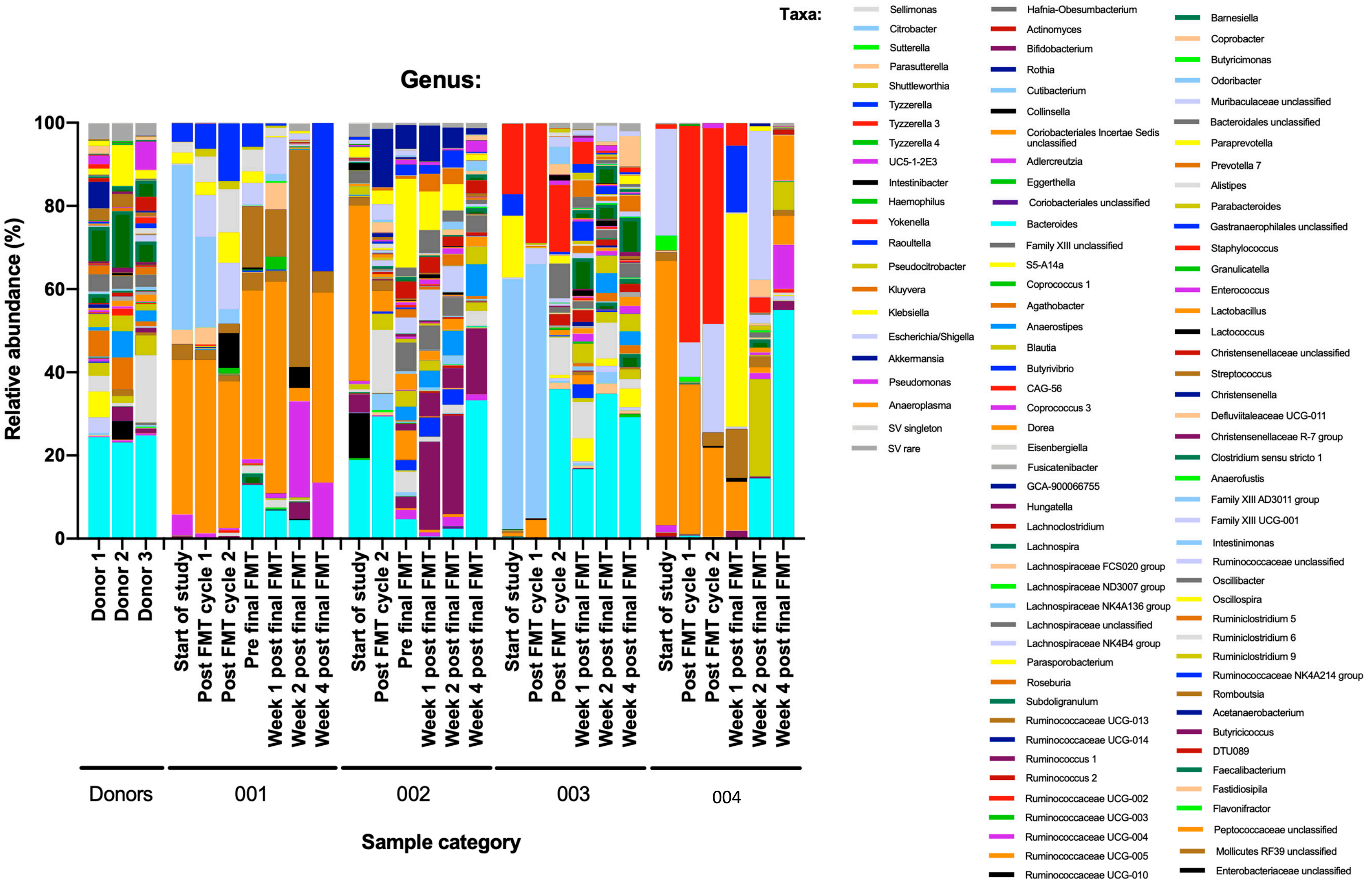
Genus:
Relative abundance (%)
100
80
60
40
20
0
Donor 1
Donor 2
Donor 3
Start of study
Post FMT cycle 1
Post FMT cycle 2
Pre final FMT
Week 1 post final FMT
Week 2 post final FMT
Week 4 post final FMT
Donors
001
002
003
004
Sample category
Taxa:
Sellimonas
Citrobacter
Sutterella
Parasutterella
Shuttleworthia
Tyzzerella
Tyzzerella 3
Tyzzerella 4
UC5-1-2E3
Intestinibacter
Haemophilus
Yokenella
Raoultella
Pseudocitrobacter
Kluyvera
Klebsiella
Escherichia/Shigella
Akkermansia
Pseudomonas
Anaeroplasma
SV singleton
SV rare
Hafnia-Obesumbacterium
Actinomyces
Bifidobacterium
Rothia
Cutibacterium
Collinsella
Coriobacteriales Incertae Sedis unclassified
Adlercreutzia
Eggerthella
Coriobacteriales unclassified
Bacteroides
Family XIII unclassified
S5-A14a
Coprococcus 1
Agathobacter
Anaerostipes
Blautia
Butyrivibrio
CAG-56
Coprococcus 3
Dorea
Eisenbergiella
Fusicatenibacter
GCA-900066755
Hungatella
Lachnoclostridium
Lachnospira
Lachnospiraceae FCS020 group
Lachnospiraceae ND3007 group
Lachnospiraceae NK4A136 group
Lachnospiraceae unclassified
Lachnospiraceae NK4B4 group
Parasporobacterium
Roseburia
Subdoligranulum
Ruminococcaceae UCG-013
Ruminococcaceae UCG-014
Ruminococcus 1
Ruminococcus 2
Ruminococcaceae UCG-002
Ruminococcaceae UCG-003
Ruminococcaceae UCG-004
Ruminococcaceae UCG-005
Ruminococcaceae UCG-010
Barnesiella
Coprobacter
Butyricimonas
Odoribacter
Muribaculaceae unclassified
Bacteroidales unclassified
Paraprevotella
Prevotella 7
Alistipes
Parabacteroides
Gastranaerophilales unclassified
Staphylococcus
Granulicatella
Enterococcus
Lactobacillus
Lactococcus
Christensenellaceae unclassified
Streptococcus
Christensenella
Defluviitaleaceae UCG-011
Christensenellaceae R-7 group
Clostridium sensu stricto 1
Anaerofustis
Family XIII AD3011 group
Family XIII UCG-001
Intestinimonas
Ruminococcaceae unclassified
Oscillibacter
Oscillospira
Ruminiclostridium 5
Ruminiclostridium 6
Ruminiclostridium 9
Ruminococcaceae NK4A214 group
Romboutsia
Acetanaerobacterium
Butyricicoccus
DTU089
Faecalibacterium
Fastidiosipila
Flavonifractor
Peptococcaceae unclassified
Mollicutes RF39 unclassified
Enterobacteriaceae unclassified

Supplementary Figure S3. Fecal metataxonomic changes in relation to sequential FMT in patients with severe or fulminant *Clostridioides difficile* infection. 16S rRNA gene sequencing of DNA extracted from stool samples, presented as relative abundance plots. Participant samples presented as: three stool donors; patient 1(001), earliest to latest timepoint; patient 2 (002), earliest to latest timepoint; patient 3 (003), earliest to latest timepoint, and; patient 4 (004), earliest to latest timepoint. **(A) Class:** Healthy donor stool microbiomes were dominated by *Bacteroidia* and *Clostridia*, and severe CDI patients successfully treated by serial FMTs consistently demonstrated an increase in the relative abundance of these classes in their stool microbiome over the course of treatment. In contrast, treatment non-responder (patient 1) did not demonstrate this progression Figure S2 and retained a stool microbiome dominated by *Bacilli* and *Gammaproteobacteria*, similar to baseline assessment. **(B) Order:** Baseline stool samples from all four patients demonstrated particularly high relative abundance of Lactobacillales and Enterobacteriales, together with the smaller but consistent presence of *Betaproteobacteriales*. Over the course of therapy in all three responders, the relative abundance of all of these orders decreased to much more modest levels, with *Bacteroidales* and *Clostridiales* replacing them to become the dominant orders within the stool microbiome; this is comparable to the microbiome composition present in all three stool donors. Conversely, no consistent change in stool microbiome composition relative to baseline was observed over the course of FMT in the non-responder (patient 1). **(C) Family:** Over the course of treatment for the non-responder (patient 1), minimal changes were seen in stool microbiome composition at family level relative to the baseline sample, with *Lactobacillaceae* and *Enterobacteriaceae* remaining the dominant families, and *Streptococcaceae* present at a smaller but consistent relative abundance. In contrast, marked changes in stool bacterial family composition were seen in the three responders, transitioning in particular towards high relative abundances of *Bacteroidaceae*, *Ruminococcaceae* and *Lachnospiraceae.* The post-FMT stool bacterial family composition in responders at the end of analysis was similar to that observed in the three healthy donors. **(D) Genus:** Baseline samples from these patients were characterised by prominent relative abundances of genera including Lactobacillus and Citrobacter. Over the course of therapy in the three responders, there was restoration of genera including *Bacteroides, Faecalibacterium* and *Bifidobacterium*, again resulting in stool microbiome profiles more comparable to healthy stool donors. In contrast, only much more modest increases were seen in these particular genera over the course of therapy in the non-responder

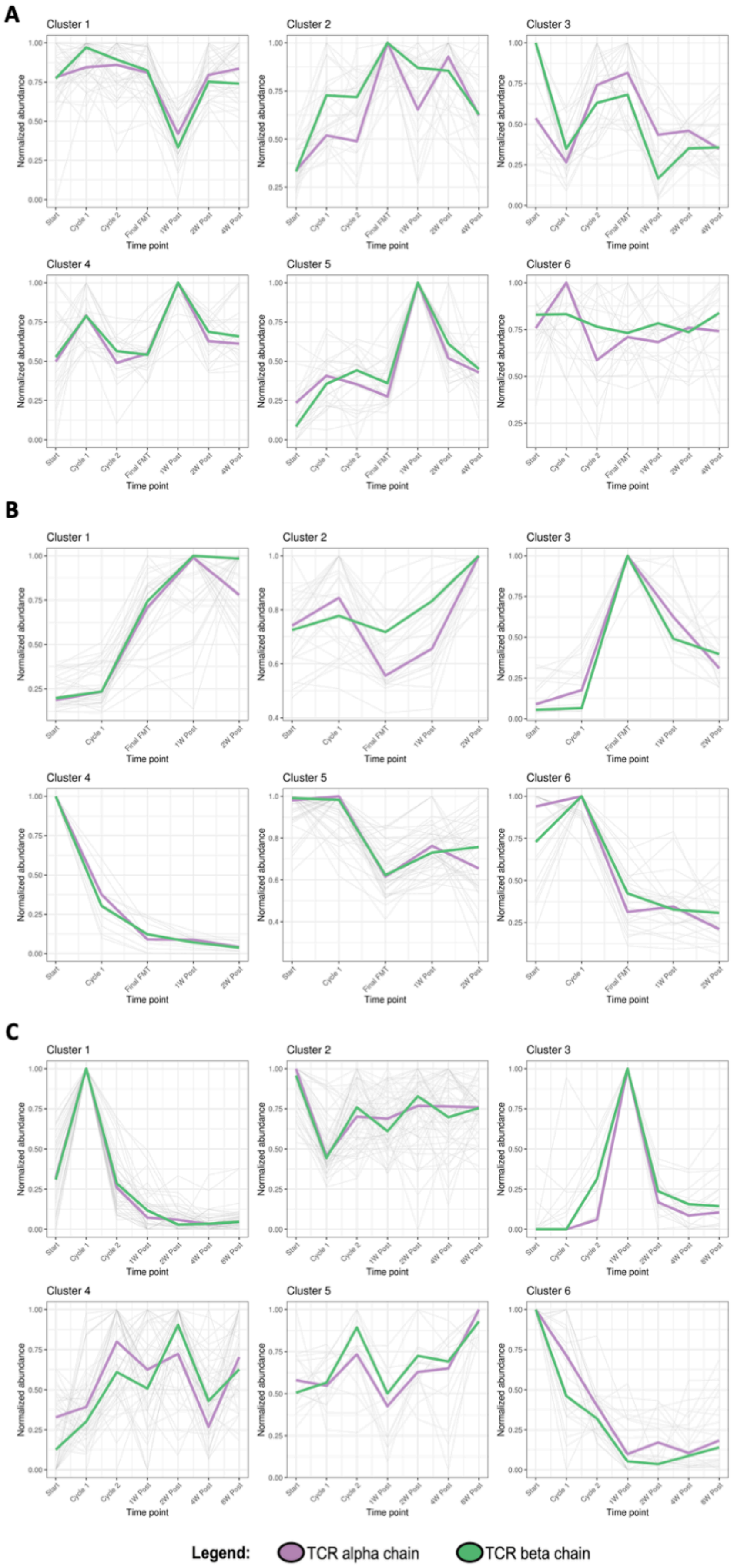

Supplementary Figure S4. Temporal clustering of most abundant TCRs for FMT responders and non-responder. Mfuzz based TCR temporal clustering. The most abundant 50 TCR clonotypes for each patient and time point were considered, (A) Patient 1 (P1), FMT failure (F)/non-responder. The same analysis is presented for (B) Patient 2 (P2), FMT success (S)/responder and (C) Patient 3 (P3), FMT success (S)/responder.

A

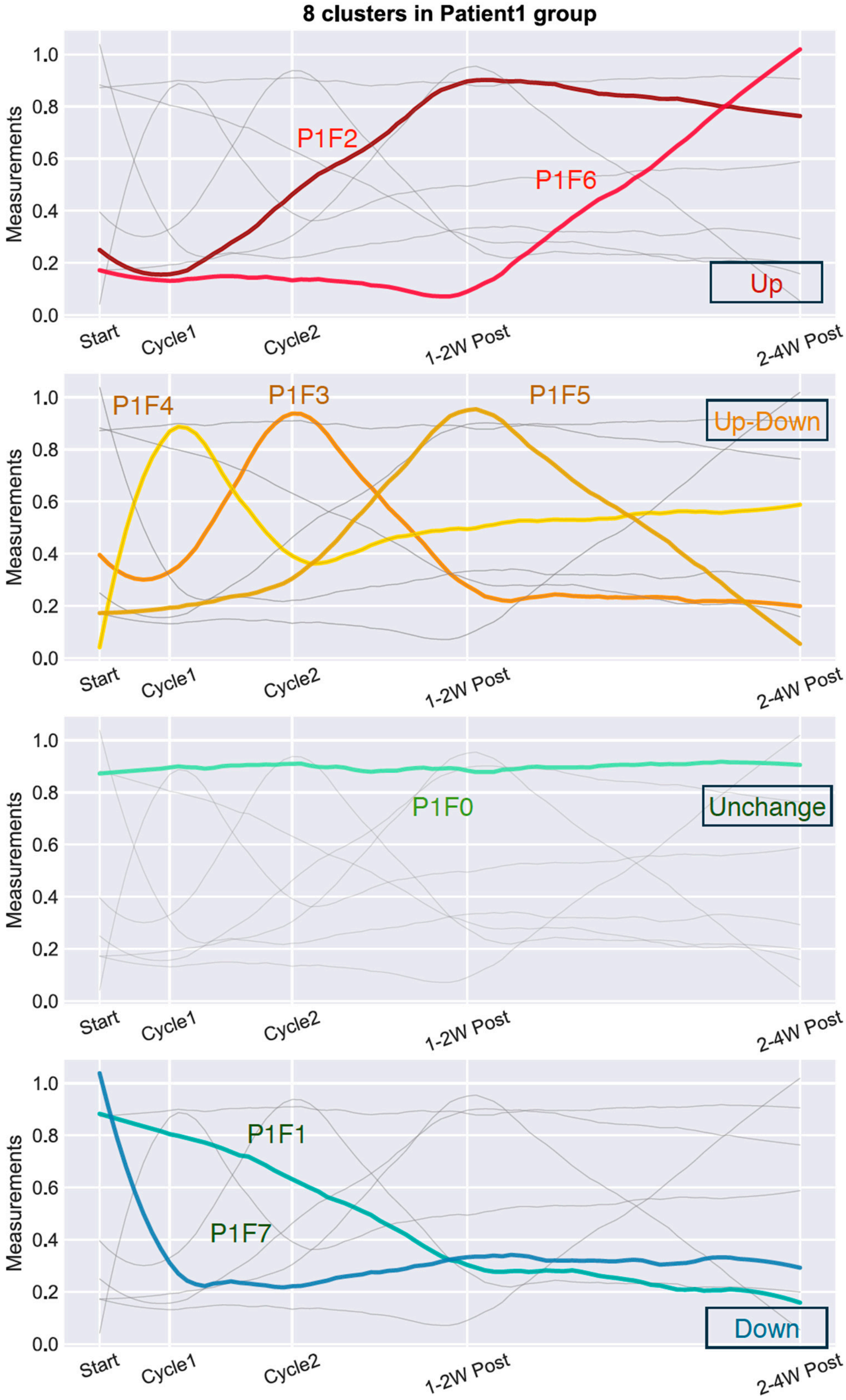
8 clusters in Patient1 group
Measurements
1.0
0.8
0.6
0.4
0.2
0.0
Start
Cycle1
Cycle2
1-2W Post
2-4W Post
P1F2
P1F6
Up
P1F4
P1F3
P1F5
Up-Down
P1F0
Unchange
P1F1
P1F7
Down

B

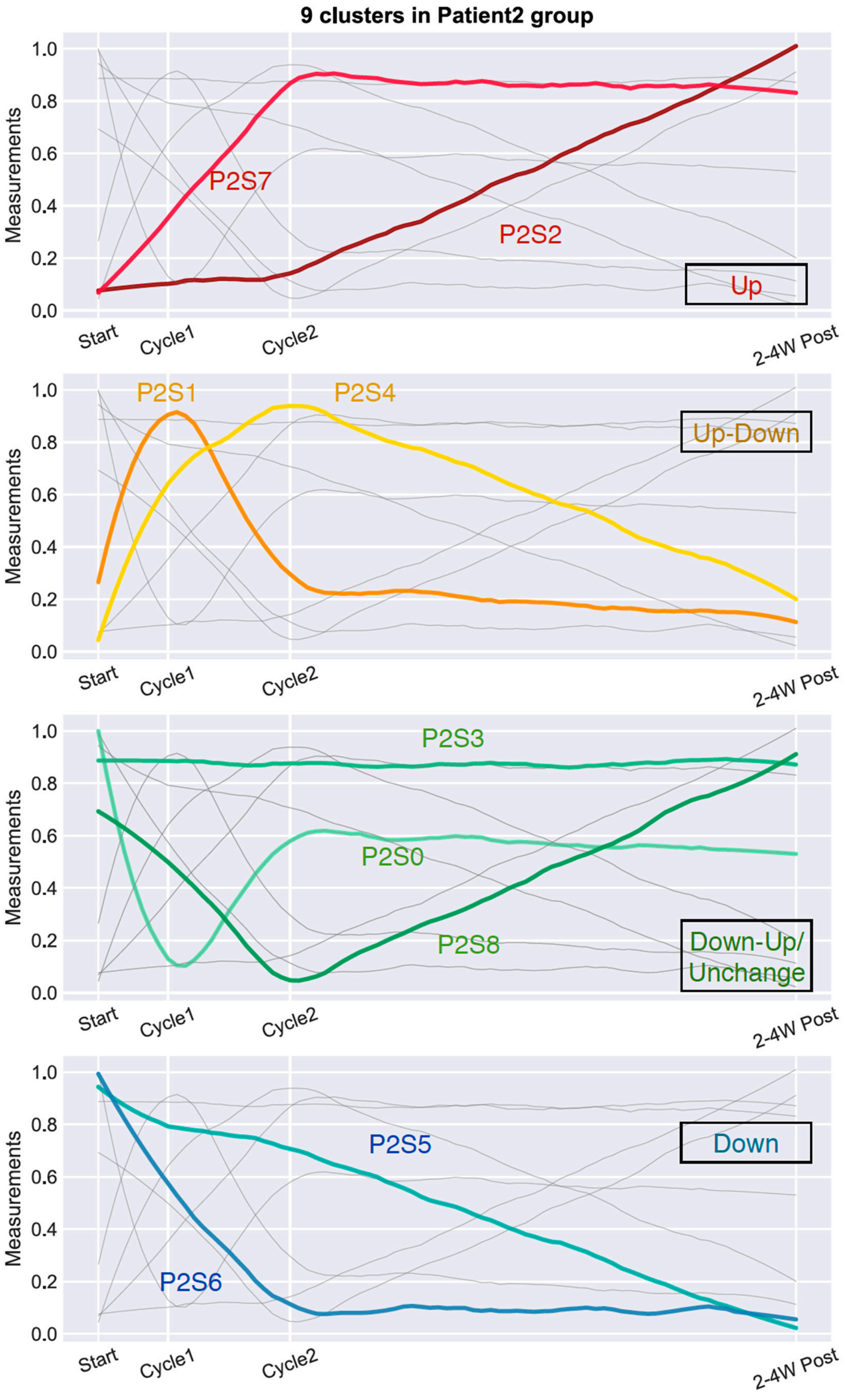

9 clusters in Patient2 group
Measurements
1.0
0.8
0.6
0.4
0.2
0.0
Start
Cycle1
Cycle2
2-4W Post
P2S7
P2S2
Up
P2S1
P2S4
Up-Down
P2S3
P2S0
P2S8
Down-Up/
Unchange
P2S5
P2S6
Down

C

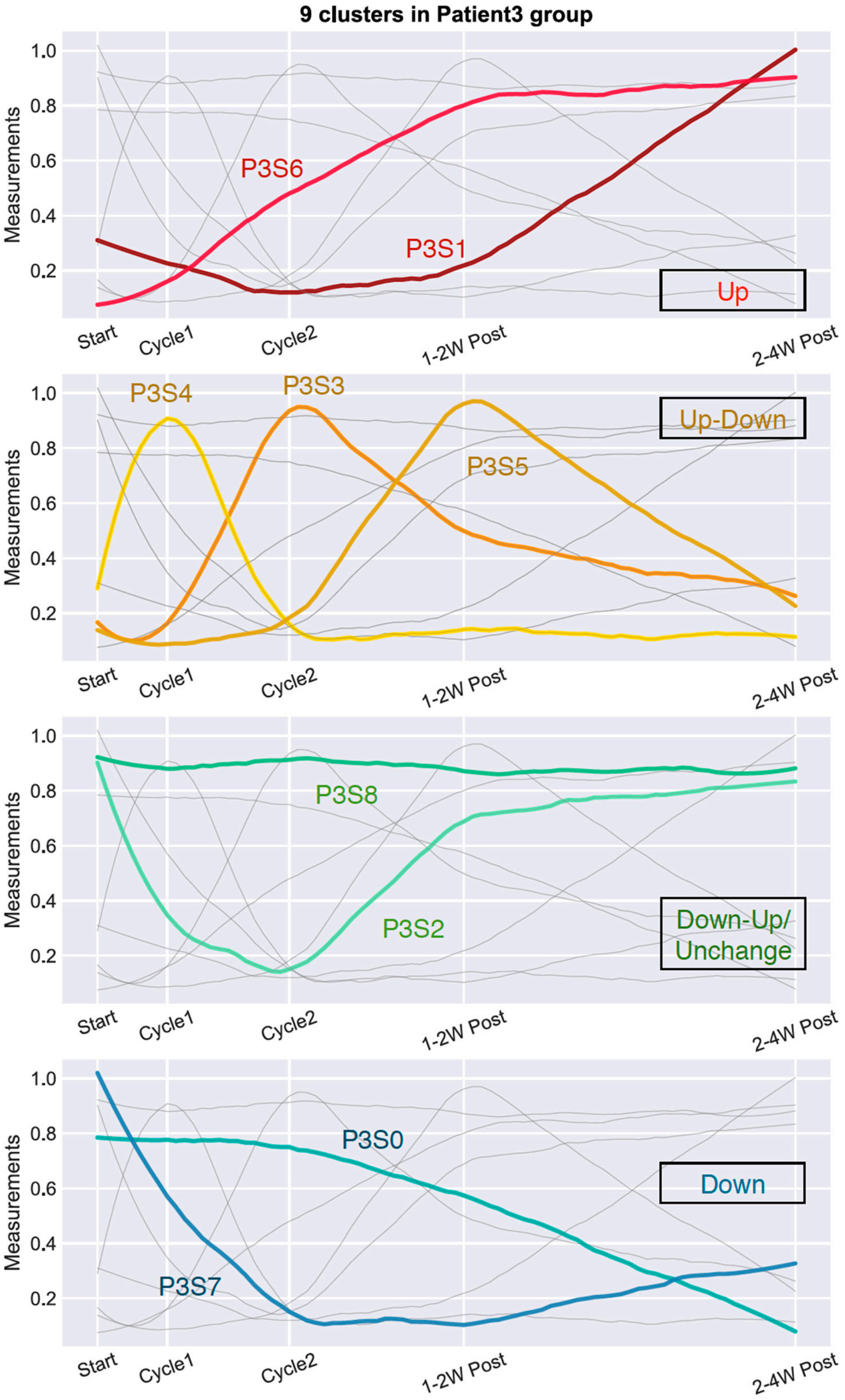

Supplementary Figure S5. K-means integrative clustering of all measures including TCR alpha and beta clonotypes for FMT responders and non-responder. Patient 1 (P1), FMT failure (F)/non-responder. (B) Patient 2 (P2), FMT success (S)/responder with each cluster indexed (C) Patient 3 (P3), FMT success (S)/responder with each cluster indexed. Clusters are based on the temporal behaviour of features over 5 time points. Clusters are re-grouped into four categories: increased after FMT (Up, red), increased after FMT but recovered (Up-Down, yellow), decreased after FMT but recovered (Down-Up) or Unchanged, green,

and decreased after FMT (Down, blue). Features within each indexed cluster for patients 1-3 are presented in Tables S5a-c, respectively.

Supplementary Table S1. Dictionary of longitudinal features assessed in multiomics study (n=681)

[Uploaded separately as Excel file due to size]

Supplementary Table S2. Convergent features identified for FMT responders and non-responder

Feature	Feature Type	Start higher in	Fold Change log2 (succ/fail) pre-FMT	p-value (pre-FMT)	Fold Change log2 (succ/fail) post-FMT	p-value (post-FMT)
A002 IgG	Antigen-specific antibody panel	Success	2.168447383	0.021598466	0.076664339	0.912891473
CD4 EMRA(%)	Flow Cytometry	Success	1.761077742	0.016785885	-0.120622559	0.708534909
miR-23a-3p	Serum MicroRNA	Success	1.201615131	0.040607084	0.437550391	0.579171863
CD4:CD8 T cell ratio	Flow Cytometry	Success	1.197220744	0.004186219	1.221598649	0.068527041
Taurodeoxycholic Acid-3-Sulfate	Stool Bile Acids	Success	1.072140319	0.027285868	-0.354160177	0.699011958
Glycodeoxycholic acid	Stool Bile Acids	Success	0.952510319	0.041095589	0.492763241	0.373079466
CD28 expression levels on CD4 T cells (MFI)	Flow Cytometry	Success	0.886320797	0.040484759	0.921093359	0.05346842
IL4 expression levels in stimulated CD4 T cells (MFI)	Flow Cytometry	Success	0.586016717	0.031999166	1.796466606	0.196646558
IgGII1H5N4F1S1; IgG2&3 glycopeptide with digalactosylated and monosialylated glycan with core fucose	IgG Glycoprofiling	Success	0.437264647	0.017212106	0.251641259	0.459770001
IL4+ve stimulated CD4 T cells (%)	Flow Cytometry	Success	0.350398491	0.020802347	0.366496604	0.097774347
Senescent NKG2D expression levels on CD4 T cells (MFI)	Flow Cytometry	Success	0.257360075	0.003461509	0.161912393	0.070534502
Senescent NKG2D expression levels on CD4 T cells (MFI)	Flow Cytometry	Success	0.208923649	0.008213432	0.132820495	0.137849534
Digalactosylated glycans	Serum glycan traits	Success	0.139048518	0.015584596	0.15869462	0.130148886
TWEAK/TNFSF12	Inflammation panel	Failure	-0.20312782	0.005215789	-0.112393335	0.196678529
IgGII1H3N4; IgG2&3 glycopeptide with agalactosylated glycan without core fucose	IgG Glycoprofiling	Failure	-0.36672315	0.041648577	-0.270400717	0.072381241
Chitinase 3-like 1	Inflammation panel	Failure	-0.369364293	0.025410356	-0.168913433	0.623049192
IgGII1H4N4; IgG2&3 glycopeptide with monogalactosylated glycan without core fucose	IgG Glycoprofiling	Failure	-0.377672881	0.04057148	-0.156220857	0.467559648
T cells (%)	Flow Cytometry	Failure	-0.389587322	0.000805873	-0.035851059	0.812428456
CD57 expression levels on CD4 T cells (MFI)	Flow Cytometry	Failure	-0.431826795	0.038188962	0.658496274	0.093403683
Cholic Acid-3-Sulfate	Stool Bile Acids	Failure	-0.531041071	0.005367387	-0.205555769	0.658217617
VP1 Tox B IgA	Antigen-specific antibody panel	Failure	-1.176152397	0.028922776	-0.524567556	0.434758869
VP1 ToxA IgG	Antigen-specific antibody panel	Failure	-1.243666984	0.025030736	-0.251258925	0.845753324
8(14),(5-beta)-Cholenic Acid-3-alpha, 12-alpha-diol	Stool Bile Acids	Failure	-1.32053665	0.015044042	-0.119199539	0.843419393
Raoultella	Genus	Failure	-1.531677864	0.031308584	-0.672727411	0.773643733
3-alpha-Hydroxy-7,12-DiketoCholanic Acid	Stool Bile Acids	Failure	-1.742191231	0.032820762	-0.926287754	0.396171749
Enterococcaceae	Family	Failure	-1.74757102	0.02667642	-1.973860785	0.097431927
Enterococcus	Genus	Failure	-1.74757102	0.02667642	-1.973860785	0.097431927
Enterobacteriaceae unclassified	Genus	Failure	-2.227394233	0.000830761	-2.154577971	0.260908653
Pseudocitrobacter	Genus	Failure	-2.238925946	0.016836699	-2.110493797	0.270525836

Supplementary Table S3. Divergent features identified for FMT responders and non-responder

Feature	Feature Type	Fold Change log2 (succ/fail) pre-FMT	p-value (pre-FMT)	Fold Change log2 (succ/fail) post-FMT	p-value (post-FMT)	Finish higher in
Bregs (%)	Flow Cytometry	1.68975869	0.06469277	3.12772671	0.00185911	Success
IL4 expression levels in stimulated CD4 T cells (MFI)	Flow Cytometry	0.31557843	0.44291517	1.27759122	0.00950044	Success
Acetate (calc. conc)	Stool SCFA	-0.1673595	0.33176535	0.99613672	0.00411013	Success
Lithocholic acid	Stool Bile Acids	0.05104188	0.91379976	0.99174456	0.04336816	Success
CD28 expression levels on CD8 T cells (MFI)	Flow Cytometry	0.18736624	0.35575933	0.5557157	0.04837418	Success
Acetate (ug/g)	Stool SCFA	-0.0865381	0.33421227	0.47227339	0.0037415	Success
IgGII1H4N4F1; IgG2&3 glycopeptide with monogalactosylated glycan with core fucose	IgG Glycoprofiling	0.29200766	0.10481597	0.45427218	0.0289065	Success
Low-branching glycans	Serum glycan traits	0.09290351	0.17146535	0.1389985	0.03391767	Success
IgGII1H4N4S1; IgG2&3 glycopeptide with monogalactosylated and monosialylated glycan without core fucose	IgG Glycoprofiling	-0.1137404	0.21350884	-0.0961381	0.04114768	Failure
Total memory CD8 T cells (%)	Flow Cytometry	-0.1381691	0.06566426	-0.2388483	0.00385259	Failure
IgGI1H4N5F1; IgG1 glycopeptide with bisected monogalactosylated glycan with core fucose	IgG Glycoprofiling	0.02136881	0.86872911	-0.2453512	0.02330967	Failure
IgGII1H4N5S1; IgG2&3 glycopeptide with bisected monogalactosylated and monosialylated glycan without core fucose	IgG Glycoprofiling	-0.2297707	0.25951037	-0.2786098	0.01431667	Failure
IgGI1H4N5F1S1; IgG1 glycopeptide with bisected monogalactosylated and monosialylated glycan with core fucose	IgG Glycoprofiling	-0.0079357	0.92809602	-0.3236003	0.0322431	Failure
Senescent CD57+ve CD8 T cells (%)	Flow Cytometry	-0.3562092	0.1470277	-0.32671	0.01498308	Failure
Trisialylated glycans	Serum glycan traits	-0.2363198	0.19608774	-0.3654888	0.04019557	Failure
High-branching glycans	Serum glycan traits	-0.2344961	0.1397511	-0.3942921	0.01229867	Failure
Senescent CD57+ve CD4 T cells (%)	Flow Cytometry	-0.0870038	0.72407094	-0.567451	0.0197068	Failure
sTNF-R2	Inflammation panel	-0.6969736	0.06935009	-0.6998909	0.03878925	Failure
Senescent CD28-ve CD4 T cells (%)	Flow Cytometry	-0.0914494	0.77865461	-0.7292478	0.01552293	Failure
Tetragalactosylated glycans	Serum glycan traits	-0.2336985	0.1027724	-0.7322653	0.00095655	Failure
Tetrasialylated glycans	Serum glycan traits	-0.1890873	0.22635387	-0.7346522	0.00200455	Failure
Senescent CD28-veCD57+vet CD4 T cells (%)	Flow Cytometry	-0.2027912	0.53254705	-0.7506846	0.01353224	Failure
Antennary fucosylation	Serum glycan traits	-0.3708882	0.07418547	-0.7876227	0.0057968	Failure

Supplementary Table S4. K-means cluster analysis without TCR repertoire data showing features within each indexed cluster for A. FMT Responders; B. FMT non-responder

[Uploaded separately as Excel file due to size]

Supplementary Table S5. K-means cluster analysis with TCR repertoire data showing features within each indexed cluster for A. Patient 1; B. Patient 2; and C. Patient 3.

[Uploaded separately as Excel file due to size]

Supplementary Table S6. Immunosenescent T cell parameter correlations with omics features for FMT responders and non-responder (Absolute value of Spearman Correlation Coefficient >0.9; p<0.05)

	**FMT Responders: senescent T cell correlations with omics**		**FMT Non-responder: senescent T cell correlations with omics**	
	**Positive correlation**	**Negative correlation**	**Positive correlation**	**Negative correlation**
Fecal SCFA	2-hydroxybutyrate	Valerate<br>Isobutyrate<br>2-methylbutyrate<br>Isobutyrate<br>Isovalerate<br>Butyrate<br>Propionate	Butyrate	NA
Serum SCFA	NA	Propionate<br>2-methylbutyrate	2-hydroxybutyrate	Lactate
Fecal BA	Glycoursodeoxycholic acid-3-Sulfate<br>Tauroursodeoxycholic acid<br>Glycoursodeoxycholic acid<br>Taurodeoxycholic acid-3-sulfate<br>Ursodeoxycholic acid-3-sulfate<br>Glycolithocholic acid-3-sulfate<br>Glycodeoxycholic acid-3-sulfate<br>Glycodeoxycholic acid<br>5-cholenic acid-3-beta-ol<br>3-alpha-hydroxy-7 ketolithocholic acid<br>Glycochenodeoxycholic acid<br>Taurolithocholic acid-3-sulfate<br>Glycohyocholic acid<br>Taurochenodeoxycholic acid<br>Glycochenodeoxycholic acid-3-sulfate	Hyodeoxycholic acid<br>3-alpha-hydroxy-12 ketolithocholic acid<br>Deoxycholic acid<br>Ursodeoxycholic acid<br>Isodeoxycholic acid<br>5-beta-cholanic acid-3-beta, 12-alpha-diol<br>Lithocholic acid<br>3,6-diketocholanic acid/3,12-diketocholanic acid	Ursodeoxycholic acid<br>Isodeoxycholic acid<br>Hyodeoxycholic acid	NA
Serum *N*-glycans	Trisialylated<br>High-branching<br>Trigalactosylated<br>Tetrasialylated<br>Tetragalactosylated<br>Disialylated<br>Antennary Fucosylation	Oligomannose<br>Bisection (glycans with bisecting GlcNAc)<br>Neutral (not sialylated)<br>Agalactosylated glycans<br>Core fucosylation<br>Monogalactosylated<br>Low-branching	NA	NA
Serum IgG Fc *N*-glycopeptides	IgG4H4H5<br>IgG1H4N5F1<br>IgG2H4N5F1<br>IgG2H4N5F1S1<br>IgG1H4N5F1S1<br>IgG1H4N5F1<br>IgG4H4N4F1<br>IgG4H5N4F1S1<br>IgG2H4N5F1S1<br>IgG1H4N4F1<br>IgG4H4N4F1S1<br>IgG1H4N5F1	IgG1H3N4<br>IgG4H5N4S1<br>IgG1H4N4S1	Ig4H4N5F1<br>IgG2H3N4<br>IgG2H5N5F1<br>IgG2H3N4<br>IgG4H3N4<br>IgG1H3N5F1<br>IgG4H3N5F1	IgG4H5H5<br>IgG4H5N4F1
Fecal microbiota	*Collinsella*<br>*Prevotella_7*<br>*Tyzzerella_4*<br>Coriobacteriaceae<br>*S5-A14a*<br>*Barnesiella*<br>*Ruminococcus_1*<br>*Bacilli*<br>*Lactobacillales*<br>*Mollicutes_RF_unclassified*<br>*Mollicutes_RF39_unclassified*<br>*Mollicutes RF39*<br>*Cutibacterium*<br>*Propionibacteriales*<br>*Lactobacillaceae*<br>*Lactobacillus*<br>*Sutterella*<br>*Citrobacter*	*Ruminococcaceae_UCG-004*<br>*Christensenellaceae*<br>*Faecalibacterium*<br>*Coriobacteriales_Incertae_Sedis*<br>*Coriobacteriales_Incertae_Sedis_unclassified*<br>*Christensenellaceae_R-7_group*<br>*Ruminococcaceae*<br>*Coprococcus_3*<br>*Parasporobacterium*<br>*Bifidobacteriaceae*<br>*Bifidobacteriales*<br>*Bifidobacterium*<br>*Family_XIII_AD3011_group*<br>*Family_XIII*<br>*Actinobacteria*<br>*Erysipelotrichaceae_UCG-003*<br>*Prevotellaceae*	*Pseudomonas* at the genus, family, and order level<br>*Coprococcus_1*<br>*Ruminococcaceae_014*<br>*Solibacterium*<br>*Mollicutes*<br>*Mollicutes RF39*<br>*Mollicutes_RF39_unclassified*<br>*Mollicutes RF_39_unclassifed*<br>*Rikenellaceae*<br>*Tenericutes*<br>*Alistipes*<br>*Acidaminococcaceae*	NA

	Coriobacteria<br>*Escherichia/Shigella*<br>*Coriobacteriaceae*<br>*SV_singleton*	*Paraprevotella*<br>*Defluvitaleaceae_UCG-011*<br>*Defluvitaleaceae*<br>*Christensenellaceae_unclassified*<br>*Ruminococcaceae_unclassified*<br>*Anaerofustis*<br>*Eubacteriaceae*<br>*Clostridiales*<br>*Clostridia*<br>*Lachnospiraceae_unclassfied*<br>*Erysipelotrichaceae_unclassified*<br>*Eisenbergiella*<br>*Candidatus_Stoquefichus*<br>*Butyriciococcus*<br>*Lachnoclostridium*<br>*Ruminococcus_2*<br>*Tyzzerella_3*<br>*Prevotellaceae*<br>*Ruminiclostridium_6*<br>*Faecalitalea*<br>*Subdoligranulum*<br>*Lachnospiraceae*<br>Ruminococcaceae	*Phascolarctobacterium*	
Inflammation-related proteins	sTNF-R1<br>IL-29/IFN-lamda1<br>sTNF-R2<br>Osteopontin<br>LIGHT/TNFSF14<br>IL-11<br>Pentraxin-3<br>IFN-γ<br>MMP-1<br>IL-28A/IFN-lamda2<br>sIL-6Ra<br>TWEAK/TNFSF12<br>IL-2<br>IL-10<br>IL-12 (p40)<br>IL-26<br>IFN-a2<br>IL-27<br>sCD30/TNFRSF8<br>IL-32<br>TSLP<br>IFN-b<br>IL-35<br>IL-19<br>sCD163<br>IL-8	NA	Osteocalcin	MMP-1<br>Osteopontin<br>IL-26<br>Chitinase 3-like 1
Serum Ig	EBV IgA	IgM antibodies to SLPs of *C. difficile* ribotypes 001, 027<br>CMV IgM<br>Tetanus IgG<br>Toxin A IgG<br>Total IgM, IgA, IgG1, IgG2, IgG3, IgG4	Toxin B IgG<br>EBV IgA<br>IgG lysate 027 ribotype<br>IgG SLP 002 ribotype<br>EBV IgM<br>IgG SLP 002 ribotype<br>Toxin B IgA<br>IgM SLP 002 ribotype<br>IgA lysate 001 ribotype<br>IgG SLP 002 ribotype	Tetanus IgG<br>CMV IgG<br>IgM SLP 027 ribotype
Serum microRNA	NA	miR-23a-3p<br>miR-451a<br>Let-7b	NA	NA

Senescent T cell parameters [CD28^{-ve} CD57^{+ve} CD4 T cells (%), CD28^{-ve} CD57^{+ve} CD8 T cells (%), CD28^{-ve} CD8 T cells (%), CD28^{-ve} T cells (%), CD57^{+ve} CD8 T cells (5), CD57^{+ve} CD4 T cells (%), and CD28^{-ve} CD4 Tcells (%)] were correlated with omics features.

IgG4H4H5 = IgG4 glycopeptide with bisected monogalactosylated glycan without core fucose; IgG1H4N5F1 = IgG1 glycopeptide with bisected monogalactosylated glycan with core fucose; IgG2H4N5F1 = IgG2&3 glycopeptide with bisected monogalactosylated glycan with core fucose; IgG2H4N5F1S1 = IgG2&3 glycopeptide with bisected monogalactosylated and monosialylated glycan with core fucose; IgG1H4N5F1S1 = IgG1 glycopeptide with bisected monogalactosylated and monosialylated glycan with core fucose; IgG1H4N5F1 = IgG1 glycopeptide with bisected monogalactosylated glycan with core fucose; IgG4H4N4F1 = IgG4 glycopeptide with monogalactosylated glycan with core fucose; IgG4H5N4F1S1 = IgG4 glycopeptide with digalactosylated and monosialylated glycan with core fucose; IgG2H4N5F1S1 = IgG2&3 glycopeptide with bisected monogalactosylated and monosialylated glycan with core fucose; IgG1H4N4F1 = IgG1 glycopeptide with monogalactosylated glycan with core fucose; IgG1H3N4 = IgG1 glycopeptide with agalactosylated glycan without core fucose; IgG4H5N4S1 = IgG4 glycopeptide with digalactosylated and monosialylated glycan without core fucose; IgG1H4N4S1 = IgG1 glycopeptide with monogalactosylated and monosialylated glycan without core fucose; Ig4H4N5F1 = IgG4 glycopeptide with bisected monogalactosylated glycan with core fucose; IgG2H3N4 = IgG2&3 glycopeptide with agalactosylated glycan without core fucose; IgG2H5N5F1 = IgG2&3 glycopeptide with bisected digalactosylated glycan with core fucose; IgG2H3N4 = IgG2&3 glycopeptide with agalactosylated glycan without core fucose; IgG4H3N4 = IgG4 glycopeptide with agalactosylated glycan without core fucose; IgG1H3N5F1 = IgG1 glycopeptide with bisected agalactosylated glycan with core fucose; IgG4H5H5 = IgG4 glycopeptide with bisected digalactosylated glycan without core fucose; IgG4H5N4F1 = IgG4 glycopeptide with digalactosylated glycan with core fucose; IgG4H3N5F1 = IgG4 glycopeptide with bisected agalactosylated glycan with core fucose

Supplementary Table S7. Other selected differentiating parameters and their omics correlations for FMT responders (A) and non-responder (B) (Absolute value of Spearman Correlation Coefficient >0.9; $p<0.05$)

[Uploaded separately as Excel file due to size]

REFERENCES

Moreau, N. M., Goupry, S. M., Antignac, J. P., Monteau, F. J., Le Bizec, B. J., Champ, M. M., et al. (2003). Simultaneous measurement of plasma concentrations and 13C-enrichment of short-chain fatty acids, lactic acid and ketone bodies by gas chromatography coupled to mass spectrometry. *J. Chromatogr. B. Analyt. Technol. Biomed. Life Sci.* 784, 395–403. doi:10.1016/s1570-0232(02)00827-9.

Mullish, B. H., Pechlivanis, A., Barker, G. F., Thursz, M. R., Marchesi, J. R., and McDonald, J. A. K. (2018). Functional microbiomics: Evaluation of gut microbiota-bile acid metabolism interactions in health and disease. *Methods* 149, 49–58. doi:10.1016/j.ymeth.2018.04.028.

Wolfer, A. M., Jaketmp, Gscorreia89, and Turaga, N. (2020). phenomecentre-peakPantheR. *Zenodo*. doi:10.5281/ZENODO.3776779.
